# Suboptimal response to GnRH-agonist trigger during oocyte cryopreservation: a case series

**DOI:** 10.1186/s12958-020-00614-y

**Published:** 2020-06-05

**Authors:** Miguel Russo, Kimberly Liu, Crystal Chan

**Affiliations:** 1 Division of Gynaecologic Reproductive Endocrinology and Infertility, Department of Obstetrics and Gynecology, Mount Sinai Hospital, University of Toronto, Toronto, Canada; 2 Mount Sinai Fertility, 7th floor, 250 Dundas Street West, Toronto, Ontario M5T 2Z5 Canada; 3 Samuel Lunenfeld Research Institute, Toronto, Canada

**Keywords:** Fertility preservation, Random-start controlled ovarian stimulation, Trigger medication, Suboptimal response, GnRH agonist, Luteal-phase start protocol

## Abstract

**Background:**

Random-start, controlled ovarian stimulation (COS) has advanced the field of fertility preservation, allowing patients to expedite fertility treatment and avoid further delays to their cancer therapy. This novel approach allows patients to initiate ovarian stimulation at any point, regardless of where they are in their menstrual cycle. Luteal-phase start (LPS) protocols describe treatment cycles where COS is initiated during the luteal-phase of the menstrual cycle. LPS protocols have not been studied or optimized to the same degree as conventional, early-follicular COS. Particularly, there is a paucity of evidence evaluating treatment outcomes using different trigger medications in LPS protocols. The present study aims to evaluate the efficacy of using a GnRH agonist (GnRH-a) trigger in patients undergoing oocyte cryopreservation in LPS protocols.

**Methods:**

This descriptive case series describes two patients, recently diagnosed with cancer, who underwent oocyte cryopreservation using an LPS protocol and a GnRH-a trigger at a university-affiliated, academic center.

**Results:**

The patients described in our case series both failed to adequately respond to a GnRH-a trigger, based on their serum levels of luteinizing hormone (LH) and progesterone 12 h after their GnRH-a trigger. They both required a single rescue dose of human chorionic gonadotropin (hCG).

**Conclusions:**

These findings highlight the potential risk of a suboptimal response to a GnRH-a trigger in patients undergoing LPS, controlled ovarian stimulation for oocyte cryopreservation. This risk might be attributed to the downregulation of GnRH receptors by elevated serum progesterone levels during the luteal phase. Currently, there is insufficient evidence to recommend for or against the use of a GnRH-a trigger during LPS controlled ovarian stimulation. This case series offers a number of management strategies to mitigate this risk and emphasizes the need for further research in this area.

## Introduction

Conventionally, controlled ovarian stimulation (COS) for in-vitro fertilization (IVF) is started in the early follicular phase of the menstrual cycle. However, for cancer patients undergoing fertility preservation, this approach is being supplanted by random-start COS in order to help expedite urgent gonadotoxic chemotherapy. Random-start COS allows patients to start ovarian stimulation at any point during their menstrual cycle. Luteal phase start (LPS) is a specific type of random-start COS where patients begin stimulation in the luteal phase of their cycle, when progesterone levels are elevated.

The feasibility of random-start COS can be explained by the wave theory of follicular recruitment - a concept first described by Baerwald et al. [1] They demonstrated that two or more waves of follicle development can be recruited during a single physiologic, menstrual cycle. The efficacy of random-start COS was eventually validated by early studies demonstrating equivalent treatment outcomes when compared to conventional COS, for fertility preservation [2–4].

However, LPS protocols have not been studied or optimized to the same degree as conventional COS. There is a paucity of literature evaluating the efficacy of different trigger medications during LPS, particularly the use of a GnRH agonist (GnRH-a) to reduce the risk of ovarian hyperstimulation syndrome (OHSS). While reducing the risk of OHSS is critical to avoid delays to life-sparing cancer treatment, a suboptimal response to a GnRH-a trigger could lead to poor outcomes such as low oocyte maturity or empty-follicle syndrome (EFS) [5–11]. The present study explores the novel hypothesis that LPS may be a risk factor for a suboptimal response to a GnRH-a trigger.

## Methods

This is a descriptive, case series of two cycling, nulligravid patients undergoing oocyte cryopreservation (OC) at a tertiary, academic centre after a recent cancer diagnosis (see Table 1). Both patients underwent LPS COS using a GnRH antagonist protocol (Cetrotide 250 mcg daily) with recombinant FSH (r-FSH). A GnRH agonist trigger (buserelin 0.5 mg) was administered once the patients met a pre-specified criteria (≥ 3 follicles measuring ≥17 mm). The choice of trigger was based primarily on the discretion of the physician. It was common practice at our centre to routinely use a GnRH-a trigger for the majority of oncofertility cases to mitigate the risk of OHSS. Routine bloodwork was performed 12 h post-trigger medication to confirm an adequate rise in luteinizing hormone (LH) and progesterone. Both patients demonstrated a suboptimal response (low serum luteinizing hormone (LH) and/or progesterone (P4) levels) and required a rescue dose of hCG (r-hCG 250 mcg). Oocyte retrieval was performed 36 h after their rescue trigger, as per protocol. Written informed consent was obtained from each patient for research purposes and publication of their case study.

**Table 1:** Demographics and Cycle Characteristics Summary

Patient Demographics and Cycle Characteristics	Case 1	Case 2
Age (yrs)	33	35
Diagnosis	Hodgkin’s Lymphoma	Triple-negative Breast cancer
Ovarian Reserve Testing	AMH = 5 pmol/L; Random AFC = 14	No AMH; Random AFC = 11
P4 (nmol/L)^a^	6	16
Total Days of Stimulation	11	7
Total Dose of FSH (IU)	3375 IU	2000 IU
E2 on Trigger Day (pmol/L)	2690	1030
No.of Follicles > 15 mm on Trigger Day	5	4
LH (IU/L)^b^	53	3.7
P4 (nmol/L)^b^	4	5
Oocytes Retrieved	9	7
Mature Oocytes (MIIs)	8	7

^a^ Day 1 of gonadotropin stimulation

^b^ 12 h post GnRH-a trigger

## Result(s)

### Case 1: Hodgkin’s lymphoma

A 33-year-old nulligravid, single female was seen in consultation for consideration of fertility preservation after a recent diagnosis of Hodgkin’s lymphoma. She was scheduled to start a chemotherapy regimen which included Adriamycin, Bleomycin, Vinblastine and Dacarbazine (ABVD). She had slightly irregular menstrual cycles lasting between 28 to 40 days.

Her antral follicle count (AFC) was 14 and anti-mullerian hormone (AMH) level was 5 pmol/L (0.7 ng/mL). On Day 18 of her menstrual cycle, she had a leading follicle measuring 1.7 cm on her right ovary and her serum estradiol (E2) level was 350 pmol/L, luteinizing hormone (LH) was 20 IU/L and her progesterone (P4) was 3 nmol/L. These findings suggested that the patient was on the late-follicular phase. She was subsequently assessed 2 days later and her ultrasound showed a right-sided 2.1 cm corpus luteum. Furthermore, her E2 dropped to 221 pmol/L, LH was 7.2 IU/L and progesterone was 6 nmol/L, suggesting she was in the early luteal phase. She was started on 375 IU of recombinant follicle stimulating hormone (rFSH) (Puregon, Merck, Kirkland, Canada). On Day 5 of gonadotropin stimulation, she was started on a GnRH antagonist (Ganirelix 250mcg sc, Merck, Kirkland, Canada) daily. After 11 days of gonadotropin stimulation, her E2 was 2690 pmol/L, LH was 0.8 IU/L and P4 was less than 1 nmol/L. Transvaginal ultrasound (TVUS) showed 5 dominant follicles measuring ≥15 mm and she met pre-specified trigger criteria. She was triggered using 0.5 mg of buserelin acetate (Suprefact, Sanofi-aventis, Laval, Canada). Bloodwork performed 12 h post GnRH-a trigger revealed an LH of 53 IU/L but a P4 of 4 nmol/L. Based on these findings, she received a rescue dose of 250 μg of recombinant choriogonadotropin alpha (r-hCG) (Ovidrel, EMD Serono, Mississauga, Canada) and had her oocyte retrieval 36 h after her initial GnRH-a trigger. At the time of her egg retrieval, there were 9 oocytes retrieved of which 8 were in Metaphase II and cryopreserved.

### Case 2: breast Cancer

A 35-year-old nulligravid female was seen in consultation for consideration of fertility preservation after a recent diagnosis of triple negative, breast cancer (genetic testing pending). The patient was scheduled to start neoadjuvant chemotherapy using a dose dense Doxorubicin, Cyclophosphamide, and Paclitaxel (AC-PACL) as soon as possible. Her menstrual cycles were regular lasting approximately 28 days in length. Her past medical history was significant for hypothyroidism.

On the day of her consultation, she had an ultrasound which showed an AFC was 11 and a leading follicle on her left ovary measuring 1.6 cm. Her bloodwork revealed an E2 was 856 pmol/L, LH of 8.6 IU/L, and a progesterone of 3 nmol/L. She received a dose of 250 μg of recombinant choriogonadotropin alpha (r-hCG) (Ovidrel, EMD Serono, Mississauga, Canada) to hasten ovulation and started on gonadotropin stimulation 4 days later. At the start of stimulation, her E2 was 624 pmol/L, LH of 24 IU/L, and P4 of 16 nmol/L. Furthermore, her US showed a left-sided 1.4 cm corpus luteum. These findings suggested that the patient was in the luteal phase at the start of her ovarian stimulation. She was started on 250 IU of rFSH (Puregon, Merck, Kirkland, Canada) and 5 mg of letrozole daily (Femara, Novartis, Dorval, Canada). On Day 5 of gonadotropin stimulation, she was started on a GnRH antagonist (Ganirelix 250mcg sc, Merck, Kirkland, Canada) daily.

After 7 days of gonadotropin stimulation, her E2 was 1030 pmol/L, LH of 2.9 IU/L, and P4 of 5 nmol/L. Her ultrasound showed 4 follicles measuring ≥15 mm and she met pre-specified trigger criteria. She was triggered using 0.5 mg of buserelin acetate (Suprefact, Sanofi-aventis, Laval, Canada). Bloodwork performed 12 h post GnRH-a trigger revealed an LH of 3.7 IU/L but a P4 of 5 nmol/L. Based on these findings, she received a rescue dose of 250 μg of recombinant choriogonadotropin alpha (r-hCG) (Ovidrel, EMD Serono, Mississauga, Canada) and had her oocyte retrieval 36 h after her rescue trigger. During her egg retrieval, she had a total of 7 oocytes retrieved, all in Metaphase II. She had 3 oocytes cryopreserved and 4 oocytes fertilized with her partner’s sperm using conventional IVF. She had 4 cleavage-stage embryos which were cultured to Day 5. On Day 5, she had 2 viable blastocysts which were cryopreserved.

## Discussion

The present case series highlights the potential risk of a suboptimal response to a GnRH-a trigger during LPS. Failure to induce an adequate LH surge after a GnRH-a trigger can result in poor follicular luteinisation, oocyte maturation and oocyte yield [5–11]. While controversy remains over the exact definition, a suboptimal response has been previously defined as a serum LH below 15 IU/L or serum progesterone levels that fail to rise significantly 8 to 12 h post trigger [12]. This case series emphasizes the need to evaluate the use of a GnRH-a trigger in patients undergoing LPS, primarily in the context of fertility preservation, and explores the biological plausibility behind this hypothesis.

Currently, there is a paucity of data evaluating treatment outcomes using a GnRH-a trigger in patients undergoing LPS for fertility preservation. In the largest publication to date, Cakmak et al. [4] examined 144 fertility preservation cycles, using either conventional or random-start COS, and found no differences in treatment outcomes [4]. In this study, 63% (22/35) of random-start cycles began during the luteal phase. Moreover, 28.2% of random-start cycles used a GnRH-a trigger [4]; however, it is unclear whether any of these were luteal-phase starts. Therefore, it is difficult to estimate the efficacy of a GnRH-a trigger during LPS from this study.

Two case reports have also been published describing the use of a GnRH-a trigger in patients undergoing fertility preservation using random-start COS. Nayak and Wakim [3] published a cases series of 4 patients undergoing random-start COS using a GnRH-a trigger, only one of which had a LPS protocol. The authors concluded that a GnRH-a trigger effectively triggers oocyte maturation despite having an overall oocyte maturity rate of 63% (61 MIIs/96 oocytes) [3]. Ozkaya et al.^13^ also published a case report describing a patient undergoing random-start ovarian stimulation for fertility preservation after a diagnosis of Hodgkin lymphoma. Ten hours after a GnRH-a trigger, her serum LH levels were 89 mIU/mL and her serum progesterone levels rose to 9.4 ng/mL (30 nmol/L) suggesting an adequate response to her GnRH-a trigger. However, her oocyte maturity rate was 52% (17 MIIs/31 oocytes). The authors commented that, in their experience, they had observed a suboptimal LH surge post GnRH-a trigger in 1.3% (1/75) of cycles with random-start COS [13]. However, it is unclear what proportion of these cycles had LPS as patient demographics and cycle characteristics were not published.

The use of a GnRH-a trigger, as opposed to hCG, has become a common strategy to reduce the risk of OHSS in high risk patients. Compared to hCG, GnRH-a has a significantly shorter half-life, limiting the release of vasoactive peptides from granulosa cells, which are primarily responsible for the development of OHSS [14]. A GnRH-a trigger functions by stimulating an endogenous surge of LH that leads to luteinisation of ovarian follicles and activates oocyte maturation into Metaphase II. Animal studies have demonstrated that the ability of a species to respond to GnRH is based primarily on the density of GnRH receptors (GnRH-r) expressed within the gonadotropes in the pituitary gland [15], which is highest just prior to ovulation [16]. This is likely a necessary pre-requisite for the pituitary to generate an LH surge and trigger the process of ovulation. Moreover, GnRH-r expression in the pituitary gland is regulated – in part – by estrogen and progesterone. Estradiol has been shown to increase the expression and responsiveness of GnRH-r [17] while progesterone has been shown to have the opposite effect, with the lowest concentration of GnRH-r found during the luteal phase [18]. Moreover, the downregulation of GnRH-r mediated by progesterone appears to be dose- and time-dependent [19].

**Fig. 1:**
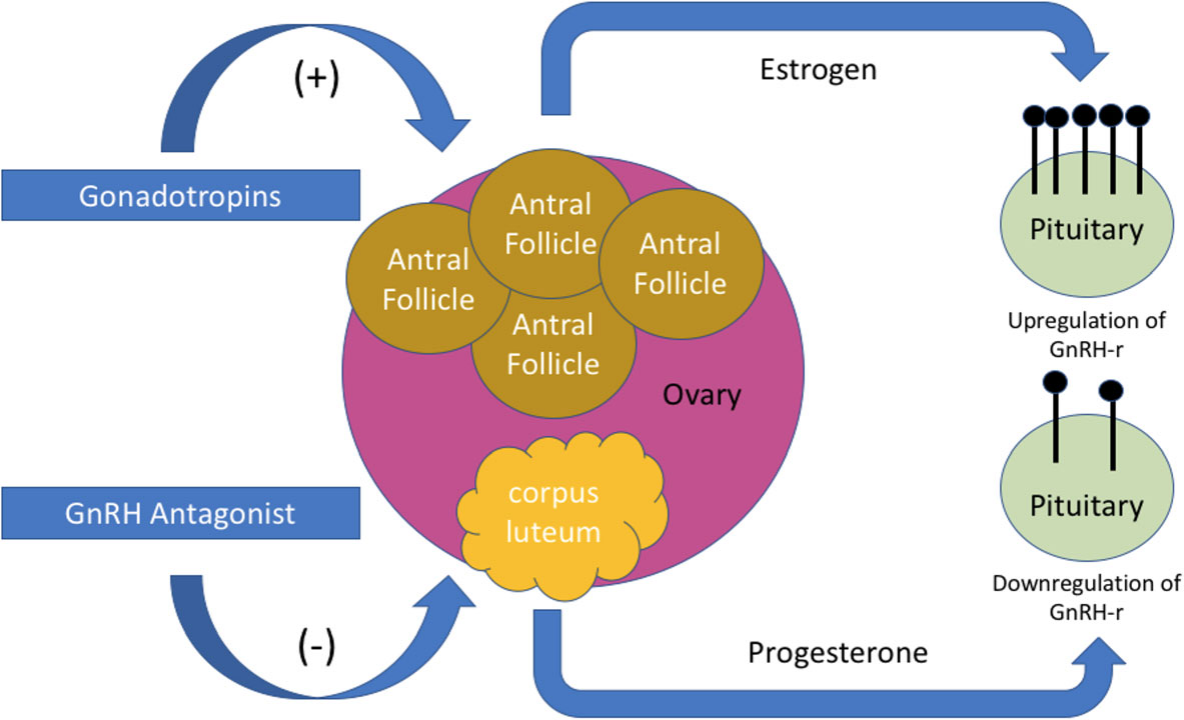
GnRH-r Regulation during Luteal-Phase COS

During LPS, the corpus luteum has already developed and production of progesterone has begun. As the elevated progesterone levels begin to downregulate GnRH-r, the rising estradiol levels from COS will begin to exert an opposite effect. Eventually, the inhibitory effect of progesterone is withdrawn by the initiation of a GnRH antagonist and resulting luteolysis. Serum progesterone levels begin to decrease as estradiol levels continue to rise, promoting upregulation of GnRH-r (see Fig. 1). However, it is plausible that – in some cases – there may be insufficient time for GnRH-r to reach the “crucial density” required to produce an optimal response to a GnRH-a trigger. Presently, this risk may be difficult to quantify given our limited knowledge about the expression of GnRH-r within humans. Likewise, it would be premature to conclude that the mere absence of “detectable” progesterone levels, as in these cases, at the time of trigger would eliminate this risk. It is possible that there might be a physiological lag time between when serum progesterone levels become undetectable and the upregulation of GnRH-r begins.

While there is limited evidence evaluating the use of a GnRH-a trigger during LPS, there are other clinical scenarios that support the risk of suboptimal response to a GnRH-a trigger in the presence of elevated progesterone levels. Several studies have been published to evaluate the efficacy of progesterone for pituitary suppression during IVF treatment [20, 21]. In a study by Kuang et al. [20], authors prospectively compared IVF outcomes between patients using medroxyprogesterone acetate (MPA) for pituitary downregulation, compared to a standard, microdose-flare protocol. For patients in the MPA group, a GnRH-a trigger was used initially; however, during the early stages of their study, the authors observed a suboptimal LH response (less than 20 IU/L) 10 h post-trigger in 5.7% of treatment cycles (3/53) [20]. These patients were subsequently found to have lower rates of oocyte maturity (0, 25 and 90%). A retrospective study was subsequently published by the same group with the intent to identify risk factors for a suboptimal response to a GnRH-a trigger (defined as an LH ≤ 15 mIU/mL) [21]. The authors examined 8960 IVF cycles with MPA or Utrogestan for pituitary downregulation, using either a GnRH-a trigger alone or in combination with hCG (1000, 2000, or 5000 IU). The rate of suboptimal response to GnRH-a trigger was 2.71% (243/8970) [21]. Patients with a suboptimal response had lower oocyte retrieval rates (48% vs 68%) and fewer mature oocytes (4 vs. 8). Compared to GnRH-a alone, the addition of hCG - irrespective of dose - was associated with an improvement in the oocyte retrieval rate but not the oocyte maturity rate [21].

In this case series, other potential causes for a suboptimal response to a GnRH-a trigger could be considered. For example, it is plausible that chronic stress due to a cancer diagnosis can lead to a state of hypogonadotropic hypogonadism which could explain a suboptimal response to GnRH-a trigger. Moreover, the suboptimal response observed between our patients was slightly different. The first patient had a suboptimal progesterone response but an adequate LH response; whereas, the second patient had suboptimal responses in both LH and progesterone levels. A possible explanation for this difference is that the second patient had significantly higher serum progesterone levels at the start of her ovarian stimulation. These findings could be attributed to the use of r-hCG to hasten ovulation prior to gonadotropin stimulation. Administration of hCG could have led to a prolonged and sustained production of progesterone from the corpus luteum, resulting in a more substantial downregulation of GnRH-r.

There are several options available to help mitigate the risk of a suboptimal response to a GnRH-a trigger, including: using an hCG trigger only for patients at low risk of OHSS; considering a “rescue trigger” using hCG if post-trigger bloodwork reveals a suboptimal response; or using a combination of a GnRH-a trigger with a low dose of hCG, commonly referred to as a “dual trigger”. While the efficacy of a dual trigger has been predominantly studied in patients undergoing IVF treatment with conventional COS, preliminary studies have been re-assuring [22, 23].

The decision on whether to use a dual trigger routinely or to give a rescue trigger only in the event of a suboptimal response is at the discretion of the physician, and depends on the patient’s individual risk of OHSS. Our report indicates that administering a rescue hCG trigger in the context of suboptimal hormonal response to GnRH-a trigger in LPS cycles can lead to an excellent yield of mature oocytes.

Timing of oocyte retrieval after suboptimal response to a GnRH-a trigger is also an important consideration. Our first patient had her egg retrieval 36 h after her initial trigger due to the adequate LH rise despite a suboptimal progesterone response. In contrast, our second patient had her egg retrieval 60 h after her initial trigger because both LH and progesterone levels failed to respond appropriately. Despite these differences in timing, both patients had successful egg retrievals. This supports the practice of delaying oocyte retrieval only if there is a suboptimal LH surge after GnRH-a trigger [12].

## Conclusion

Random-start COS has advanced the field of fertility preservation by allowing patients to start ovarian stimulation immediately, minimizing delays to their cancer treatment. However, further research is necessary to optimize treatment outcomes, such as evaluating the use of different trigger medications, particularly in the context of LPS. The present case series elucidates the plausibility of a suboptimal response to a GnRH-a trigger during LPS and provides management strategies to mitigate this risk. Currently, there is insufficient evidence to recommend for or against the use of a GnRH-a trigger alone in patients undergoing fertility preservation with LPS. Therefore, the theoretical risk of suboptimal oocyte maturation or EFS with GnRH-a trigger alone has to be balanced with the potential increased risk of OHSS by routinely using a dual trigger. In the meantime, the authors of this study recommend careful consideration when using a GnRH-a trigger alone in patients undergoing LPS, and ensuring that an adequate response is documented prior to oocyte retrieval.

## Data Availability

The datasets used and/or analysed during the current study are available from the corresponding author on reasonable request.

## Abbreviations

ABVD: Adriamycin, bleomycin, vinblastine and dacarbazine
AC-PACL: Doxorubicin, cyclophosphamide, and paclitaxel
AFC: Antral follicle count
AMH: Anti-mullerian hormone
COS: Controlled ovarian stimulation
EFS: Empty-follicle syndrome
GnRH-a: GnRH agonist
GnRH-r: GnRH receptors
hCG: Human chorionic gonadotropin
IVF: In-vitro fertilization
LH: Luteinizing hormone
LPS: Luteal-phase start
MII: Metaphase II
MPA: Medroxyprogesterone acetate
OC: Oocyte cryopreservation
OHSS: Ovarian hyperstimulation syndrome
P4: Progesterone
r-FSH: Recombinant FSH
r-hCG: Recombinant hCG
